# Biodegradable Inks in Indirect Three-Dimensional Bioprinting for Tissue Vascularization

**DOI:** 10.3389/fbioe.2022.856398

**Published:** 2022-03-25

**Authors:** Yiting Ze, Yanxi Li, Linyang Huang, Yixin Shi, Peiran Li, Ping Gong, Jie Lin, Yang Yao

**Affiliations:** ^1^ State Key Laboratory of Oral Diseases, West China Hospital of Stomatology, Sichuan University, Chengdu, China; ^2^ National Clinical Research Center for Oral Diseases, West China Hospital of Stomatology, Sichuan University, Chengdu, China

**Keywords:** biodegradable ink, indirect 3D bioprinting, vascularization, scaffold, tissue engineering

## Abstract

Mature vasculature is important for the survival of bioengineered tissue constructs, both *in vivo* and *in vitro*; however, the fabrication of fully vascularized tissue constructs remains a great challenge in tissue engineering. Indirect three-dimensional (3D) bioprinting refers to a 3D printing technique that can rapidly fabricate scaffolds with controllable internal pores, cavities, and channels through the use of sacrificial molds. It has attracted much attention in recent years owing to its ability to create complex vascular network-like channels through thick tissue constructs while maintaining endothelial cell activity. Biodegradable materials play a crucial role in tissue engineering. Scaffolds made of biodegradable materials act as temporary templates, interact with cells, integrate with native tissues, and affect the results of tissue remodeling. Biodegradable ink selection, especially the choice of scaffold and sacrificial materials in indirect 3D bioprinting, has been the focus of several recent studies. The major objective of this review is to summarize the basic characteristics of biodegradable materials commonly used in indirect 3D bioprinting for vascularization, and to address recent advances in applying this technique to the vascularization of different tissues. Furthermore, the review describes how indirect 3D bioprinting creates blood vessels and vascularized tissue constructs by introducing the methodology and biodegradable ink selection. With the continuous improvement of biodegradable materials in the future, indirect 3D bioprinting will make further contributions to the development of this field.

## Introduction

At present, only a few tissue-engineered products, such as skin (Ryssel et al., 2008) and cartilage (Makris et al., 2015), have achieved clinical success. For organs and tissues with more complex structure, such as the heart, liver, or spleen, there is still a long way to go (Berthiaume et al., 2011; Datta et al., 2017). An immature vascular system is one of the most important reasons for the failure of these products (Duan, 2017; Richards et al., 2017). In recent years, the rise of 3D bioprinting technology has enabled outstanding contributions to be made towards solving the problem of vasculature fabrication, thus extending the potential application of artificial tissues (Zhu et al., 2016; Alonzo et al., 2019; Hann et al., 2019). Indirect 3D bioprinting increasingly attracts research attention because of its superior capabilities with respect to vascularization (Wang Z. et al., 2019). Biodegradable materials are a crucial part of tissue engineering. They are typically designed to promote new tissue generation by serving as temporary templates and providing physical or chemical signals for cells (Asghari et al., 2017). Biodegradable materials also have interesting applications and new challenges in indirect 3D bioprinting for vascularization. In this review, we describe the printing methods, selection of biodegradable inks, and applications of indirect 3D bioprinting for blood vessels and vascularized tissue constructs; furthermore, we point out the existing challenges and trends for future development.

## Indirect 3D Bioprinting for Tissue Vascularization

### Vascularization Challenges in 3D Bioprinting

Vasculature is an essential part of the human body. Mature vasculature provides continuous perfusion, transporting nutrients to and removing metabolic wastes from cells, thus maintaining high cell viability and normal tissue function (Leong et al., 2013; Kim et al., 2016; Hann et al., 2019). For nonvascular tissues, the diffusion range of oxygen and nutrients is generally 100–200 μm (Carmeliet and Jain, 2000; Kaully et al., 2010). This means that, for successful *in vivo* implantation of engineered tissue constructs any larger in scale than this limit, it is necessary to ensure sufficient vasculature throughout the construct and good integration with the host vascular system (Ko et al., 2007). However, the vascularization of thick tissues has always been a major challenge and a research hotspot in tissue engineering.

3D bioprinting technology, also referred to as additive manufacturing, uses special 3D printers and bioinks containing cellular/bioactive components to fabricate scaffolds that imitate living tissues, in a layer-by-layer deposition approach with the help of computer-aided design (CAD) (Datta et al., 2017; Abdollahi et al., 2019). Compared with other tissue fabrication methods, 3D bioprinting stands out because of its convenience of customization, precise multi-dimensional control, and ability to fabricate 3D biostructures with suitable mechanical properties. In particular, its accurate control of complex and delicate structures within constructs, combined with the adoption of bioinks, make 3D bioprinting a powerful and efficient tool to address the problem of vascularization by creating a vasculogenic (Laschke and Menger, 2012; Balaji et al., 2013) (generating new blood vessels from endothelial cells or vascular progenitor cells) and angiogenic (Patel-Hett and D'Amore, 2011; Bae et al., 2012; Rouwkema and Khademhosseini, 2016) (germination or remodeling of existing vessels) environment for blood vessel formation. Most studies focus on two aspects: the fabrication of vascular grafts or vascular networks in thick constructs; however, these studies share one goal: to manufacture bionic vasculature that can work stably and continuously both *in vitro* and *in vivo*.

Although technological progress has helped the progress of vascularization in bioengineered constructs, major challenges remain (Novosel et al., 2011; Nazeer et al., 2021). The first challenge is the stable and delicate printing of microvascular networks. The diameters of capillaries, small vessels (small arteries and small veins), and small arteries with a three-layered structure are about 5–10 μm, 10–200 μm, and 30 μm, respectively; but the resolution of most 3D bioprinting is at the 100 μm level or above (Norotte et al., 2009; Hasan et al., 2014), making it difficult to print smaller diameter channels or the complex three-layered structure (adventitia, intima, and media) of blood vessels. Furthermore, the tiny hollow structure is unstable and prone to deformation or collapse owing to several factors, such as poor strength of the material, curing expansion of the bioink, or improper external extrusive force. In addition, microvascular networks should have complex multi-level branching structures. At present, the complexity of printed microvasculature hardly matches that of native vasculature (Robu et al., 2019).

The second challenge is the integration of allogeneic and autologous vascular networks. Effective integration with host circulation is the precondition of effective blood perfusion after implantation (Reis et al., 2016). Studies have shown that in implant tissue engineering, compared to the disordered vascular network formed by randomly distributed cells in culture, ordered vascular structure showed faster integration with the host and more stable perfusion, for which 3D bioprinting has the advantage (Vacanti, 2012; Baranski et al., 2013). However, the physiological blood vessel structure is so subtle that it is still difficult to use 3D bioprinting to fabricate smooth and regularly arranged microchannels at the micron level. Furthermore, most experiments are conducted *in vitro*. To date, no *in vivo* experiment has shown that, even with good integration with the host, thick tissue constructs remain viable for long or that necrosis will not occur after implantation.

Thirdly, it is difficult to ensure complex but stable blood perfusion in thick tissue constructs. Good blood perfusion is a necessary condition for engineered tissue survival *in vivo*. Currently, many strategies depend on the ingrowth of host vessels to achieve graft blood perfusion (Cheng et al., 2011; White et al., 2014; Kang et al., 2015), which is very slow (<1 mm per day) (Zhang et al., 2020), to preserve cell viability within thick tissue grafts (Sooppan et al., 2016). In order to accelerate the process, prevascularization is widely applied. Prevascularization refers to the generation of preformed microvasculature before tissue construct implantation (Laschke and Menger, 2016). It relies on seeding or encapsulating endothelial cells (ECs) in the construct *in vitro*. However, the process of cell infiltration and growth *in vitro* is uncertain, and the resultant vasculature is usually inhomogeneous (Leong et al., 2013). When implanted, immature vasculature results in insufficient blood perfusion and induces core necrosis in constructs (Asakawa et al., 2010), causing the failure of thick constructs to survive (Rouwkema et al., 2008).

Nowadays, many 3D bioprinting methods have been developed to mimic vasculature, including extrusion-, laser-based systems, electrospinning, stacking of micropatterns or modules, and cell sheet techniques (Yamamura et al., 2007; Kolesky et al., 2014; Sarker M. et al., 2018; Sarker M. D. et al., 2018). New methods are constantly being explored to solve the abovementioned problems, and indirect 3D bioprinting may cast light on vascularization in tissue engineering.

### Indirect 3D Bioprinting to Address the Problem of Tissue Vascularization

Instead of directly simulating and manufacturing the target constructs, indirect 3D bioprinting fabricates a sacrificial mold or template (negative model) and fills it with a second material. After selective dissolution, the sacrificial mold or template is removed while the construct (patrix) formed by the second material is preserved (Lee et al., 2013; Van Hoorick et al., 2015). Through this approach, pores, cavities, and channels can be precisely made throughout thick constructs (Lee et al., 2008; Schumacher et al., 2010). Owing to its specific manner of printing, other names for indirect 3D printing include lost-mold technique, indirect rapid prototyping, indirect additive manufacturing, and indirect solid free-form fabrication (Chu et al., 2001; Schumacher et al., 2010; Van Hoorick et al., 2015; Houben et al., 2016; Houben et al., 2017).

Currently, indirect 3D bioprinting has made progress in both hard and soft tissue engineering systems. Some specific tissue constructs such as functional nerve guide conduits, human knee meniscal scaffolds, sized vascular grafts, can be successfully produced in laboratories as proof of concept (Schöneberg et al., 2018; Huang et al., 2021; P B et al., 2022). At the same time, with the assistance of sacrificial molds, materials that are traditionally difficult to print can be utilized to manufacture fine structures. For example, ceramic materials play a vital role in bone tissue engineering, but its additive manufacturing is considered challenging. Chawich et al. creatively utilized a sacrificial honeycomb mold of polylactic acid (PLA), and they ultimately produced customizable stable Si-based 3D non-oxide cellular ceramic structures (El Chawich et al., 2022). Another team produced stiffness memory nanohybrid scaffolds from poly(urea-urethane) (PUU) solution which exhibits outstanding performance in long-term implantable cardiovascular devices but can not be printed directly (Wu et al., 2018). Of all the breakthroughs, the greatest concern is the potential for this technique to address problems where vascularization is a key issue.

**FIGURE 1:**
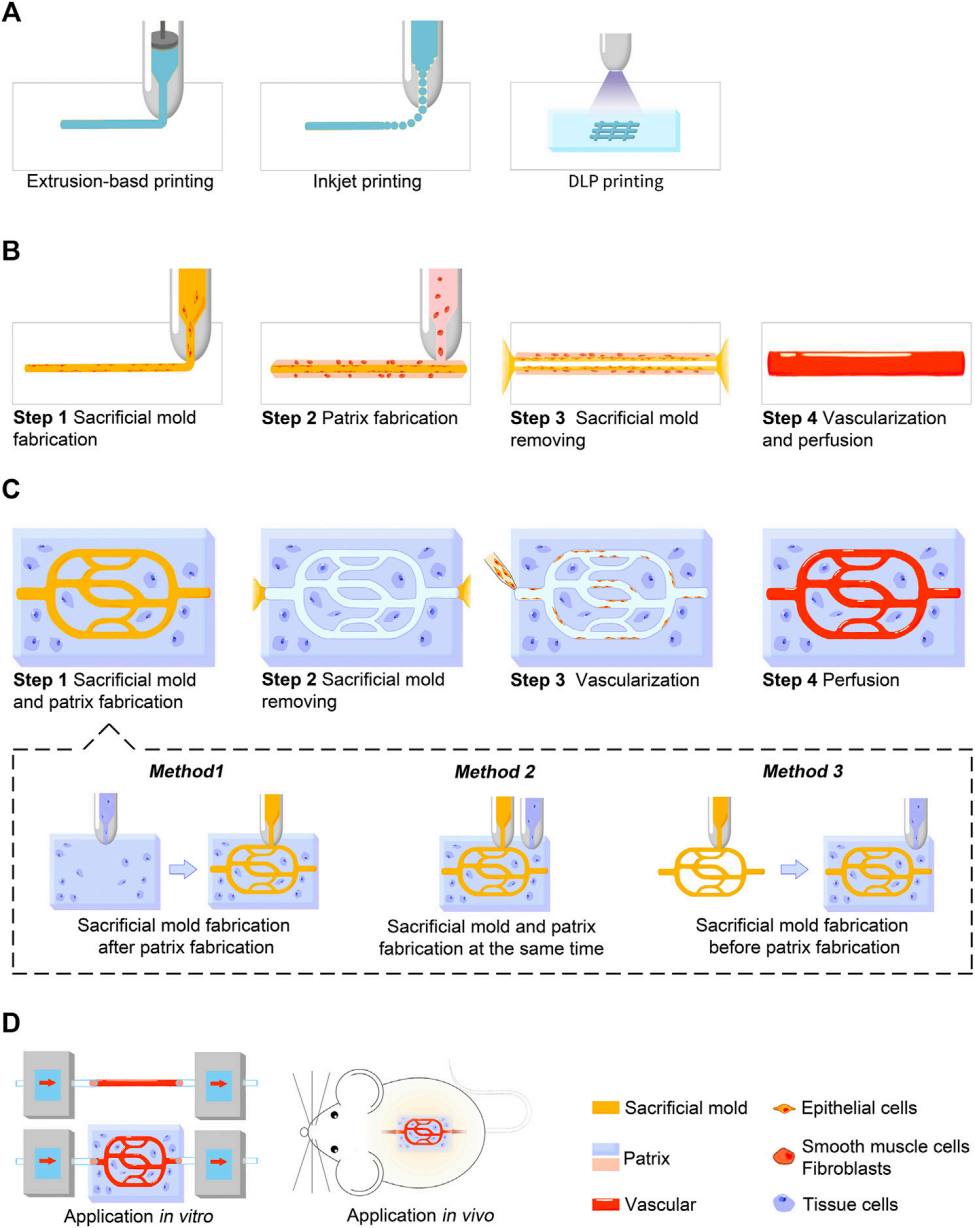
Schematic illustration showing the indirect 3D bioprinting process for blood vessels and vascularized tissue constructs. **(A)** Three major techniques used in indirect 3D bioprinting for both sacrificial mold and patrix fabrication, including extrusion-based printing, inkjet-based printing, and DLP printing. **(B)** Process of blood vessel fabrication. **(C)** Process of vascularized tissue construct fabrication. Sacrificial mold and patrix fabrication can be further divided into three methods according to the sequence of fabrication in step 1. **(D)** Applications *in vitro* or *in vivo*. Blood vessel grafts constructed by indirect 3D bioprinting are currently used for studies *in vitro*, while vascularized tissue constructs are used for both studies *in vitro* and animal experiments *in vivo*.

Generally, indirect 3D bioprinting for blood vessels and vascularized tissue constructs includes two general aspects: channel fabrication and vascularization. Channel fabrication can be further divided into three steps: sacrificial mold fabrication, patrix fabrication, and sacrificial mold removal. Manufacture of the negative mold is designed to simulate the shape and extension direction of the vascular network in a solid columnar form. In patrix fabrication, the patrix is built around the sacrificial mold to form the final scaffold or construct. Afterwards, special steps are performed to remove the sacrificial mold, leaving isometric hollow channels inside the scaffold or construct. The detailed production process and precautions of each step have been described in excellent reviews (Lee and Yeong, 2016; Houben et al., 2017; Zhu et al., 2021). Figure 1 provides a brief overview of the printing process. Here we mainly introduce the techniques frequently used and bioink selection.

The three main techniques are extrusion-based, inkjet-based, and light-assisted 3D printing (Vijayavenkataraman et al., 2018). Each technique has advantages and disadvantages, and raise different requirements for bioinks. In extrusion-based printing, the most common technique, 3D structures are built layer by layer using a nozzle to dispense continuous filament (Štumberger and Vihar, 2018; Yang et al., 2020). This technique can achieve resolutions of >100 μm, uniform cell distribution, and moderate printing speed (Placone and Engler, 2018). The technique is compatible with various materials with a viscosity range of 30 mPa s to 6 × 10^7^ mPa s (Murphy and Atala, 2014). Furthermore, its cell-friendly printing environment allows it to be used for printing scaffolds with high cell density (Skardal et al., 2015). One promising method is coaxial printing, also referred to as coaxial extrusion. Its key features are the concentric and multi-layered nozzles that enable concentric multi-material deposition. With the use of sacrificial materials, this technique allows synchronous manufacturing of the sacrificial mold and scaffold, simplifying the production process (Kjar et al., 2021). However, for extrusion-based printing, the shear stress produced during printing is an important cause of cell death and, because the nozzle size is inversely proportional to extrusion pressure, small nozzles used to improve resolution may have a greater negative impact on cell viability (Kyle et al., 2017).

Inkjet-based printing is defined as dispensing bioink through small orifices via piezoelectric, thermal, or electrostatic actuators, and accurately positioning a very small amount of the bioink on a substrate (Calvert, 2007; Datta et al., 2017). This technique can reach high resolutions of 10–100 μm with short printing times (Poellmann et al., 2011; Gudapati et al., 2016). Different inkjet printing techniques require different material viscosities. For thermal, piezoelectric, and electrostatic inkjet printing the ink viscosity should be relatively low, because the nozzle diameter is usually small and is prone to clogging (Li et al., 2020). However, for electrohydrodynamic jet bioprinting which produces droplets through an electric field, bioink with a high viscosity of over 2000 mPa s can be printed (Workman et al., 2014). Nozzle clogging, small nozzle aperture indirectly leading to cell viability damage, and low-viscosity bioink resulting in poor shape fidelity present challenges to broadening the range of implementation (Chahal et al., 2012; Malda et al., 2013; Zhu et al., 2017).

Light-assisted printing includes digital light processing (DLP)-based and laser-based printing. DLP printing is realized by dynamically projecting an entire computer-generated optical mask into a photosensitive prepolymer solution to induce photopolymerization, while laser-based printing uses a bottom-up approach (drop-by-drop) to build constructs (Zhu et al., 2021). DLP printing is now attracting greater attention as this technique provides precise control of scaffold structures and features and high printing speed (Mandrycky et al., 2016). Nevertheless, the selection of biomaterials is limited to photosensitive polymers and additional chemical modifications are required for most biomaterials (Yue et al., 2015). Moreover, the properties of printing complex patterns directly make DLP more frequently used in direct printing. Therefore, light-assisted printing has only been used in a few cases of indirect 3D printing (Thomas et al., 2020).

Another technique called embedded extrusion bioprinting involves printing a cell-containing hydrogel in a supporting bath, which serves as a sacrificial printing environment (Rocca et al., 2018). As atypical indirect 3D bioprinting, this technique does not impose any requirements for the shape of the sacrificial mold but instead skips this step and directly prints the male mold. The advantage is that low-viscosity hydrogels can be used to construct complex draping and hollow structures with the support of the sacrificial supporting bath via free-form bioprinting, which is difficult to achieve in air. Limitations are that shape fidelity of the complex pore structure is low, resolution is currently at the millimeter level, and the removal of the sacrificial supporting bath is relatively cumbersome (Afghah et al., 2020).

Table 1 summarizes the characteristics of the common techniques covered in this review.

**TABLE 1:** 3D bioprinting techniques for indirect 3D bioprinting covered in this review.

3D bioprinting techniques	Sacrificial mold or patrix fabrication	Supported bioink viscosity	Minimum feature resolution	Printing speed	Shape fidelity	Frequency of use	References
Extrusion	Sacrificial mold and patrix fabrication	Wide range of 30–6 × 10^7^ mPa s	>100 μm	Moderate	Good	+++	Murphy and Atala, (2014)
							Massa et al. (2017)
							Yang et al. (2020)
							Zou et al. (2020)
Inkjet	Sacrificial mold and patrix fabrication	Low in most cases	10–100 μm	High	Poor	+++	Yeong et al. (2007)
							Chahal et al. (2012)
							Schöneberg et al. (2018)
							Xu et al. (2018)
Light-assisted	Sacrificial mold and patrix fabrication	Low	5–300 μm	High	Good	+	Kang and Cho, (2012)
							Mandrycky et al. (2016)
							Ji et al. (2019)
							Thomas et al. (2020)
Embedded extrusion	Patrix fabrication in sacrificial supporting bath	Low to medium	At millimeter level	Slow	Medium	++	Rocca et al. (2018)
							Štumberger and Vihar, (2018)
							Afghah et al. (2020)
							Chiesa et al. (2020)

### Vascularization Strategies

Vascularization is essential for engineered tissue grafts to achieve biological function and can be realized by seeding ECs into hollow scaffold channels (Bae et al., 2012; Massa et al., 2017). After migration, survival, proliferation, and differentiation of ECs, a monolayer of cells is ultimately formed on the surface of the channels, mimicking the physiological vessel wall. Usually, growth factors and other bioactive factors are perfused along with ECs to regulate cell behavior or promote cell differentiation (Auger et al., 2013). The generation of new blood vessels involves complex effects of a variety of cells, factors released by platelets, extracellular matrix, and angiogenic and anti-angiogenic factors. Vascular endothelial growth factor (VEGF) and fibroblast growth factor b (FGFb) are the most effective angiogenic growth factors and are commonly used in the making of angiogenic biomaterials (Sun et al., 2011; Matsui and Tabata, 2012). Research reports successful seeding of human umbilical vein endothelial cells (HUVECs) into microchannels after mold sacrifice, and a uniform HUVEC layer formation coating the channels. It was demonstrated that the HUVEC layer could, to some extent, act as a barrier to protect scaffold cells from harmful substances (Massa et al., 2017). However, a potential problem is that the channel diameter may affect cell inoculation. Narrow channels will become blocked by ECs, hindering perfusion, while diameters too large will require more time for cells to cover the channel surface. Studies have shown that ideal channel diameter ranges from 280 to 1,270 μm (Zhang et al., 2016).

Another elegant vascularization strategy of indirect 3D bioprinting is to encapsulate ECs in the sacrificial ink to realize synchronous distribution of ECs in the process of blood vessel (sacrificial channel) fabrication. This approach reduces the manufacturing process and can realize precise control of cell distribution (Thomas et al., 2020). Owing to direct contact with cells and bioactive factors, biocompatibility and biodegradability of materials are particularly important. Studies have demonstrated that cells show increased proliferation and proliferation in hydrogels with faster degradation rates (Patterson and Hubbell, 2010). A further study suggests that biodegradable materials can promote angiogenesis through temporarily controlled delivery of siRNAs (Nelson et al., 2014). Application of biodegradable materials may better promote vascularization.

## Biodegradable Ink Selection

Biodegradable materials are the main scaffold materials in the field of tissue engineering, which have a broad range of applications in medicine and pharmacy because of good biosafety, reducing inflammation, ability to mimic the extracellular matrix (ECM), and enzymatic degradation *in vivo* (Asghari et al., 2017). Eventually these scaffolds are replaced by host tissues (Silva et al., 2020). Biodegradable materials are generally classified into synthetic materials and natural materials. Synthetic materials such as polyglycolic acid (PGA), polycaprolactone (PCL), PLA, poly (N-isopropylacrylamide), and their copolymers have already been widely used in tissue engineering. Advantages are that the key properties of these materials as scaffolds can be artificially controlled, such as degradation rate and some specific mechanical properties, including stiffness and elasticity; however, their biosafety is comparatively poor. Natural materials, such as chitin, alginate, collagen, and gelatin, are polymers produced by biological systems. Natural materials have many advantages and disadvantages. The most prominent advantage is their similarity to host tissues, including the ability to communicate with biological systems, enzymatic degradation, metabolic compatibility, and low inflammatory response (Asghari et al., 2017). However, owing to the poor strength of natural materials, scaffolds often collapse before finishing their task. In addition, they generally show slow and inhomogeneous degradation *in vivo*, and are inconsistent with host tissue regeneration rate.

In the field of indirect 3D printing, biodegradable polymers play vital roles in fabricating vascular or vascularized tissue: 1) As scaffold materials, they allow endothelial cells and a variety of angiogenic factors to exist, as well as providing a flexible environment for complex angiogenesis. 2) As sacrificial materials, compared with non-degradable ones, the damage to cell activity when manufacturing or removing the sacrificial molds is much lower. 3) Since the biodegradable materials can degrade *in vivo*, the use of them as sacrificial material helps to skip the processing step of sacrificial mold removal *in vitro*, thus shortening the manufacturing time. 4) In theory, by regulating and matching the degradation rate of scaffolds and sacrificial materials, the removal of sacrificial channels and channel endothelialization can be completed *in vivo* before the scaffolds completely degrade, thus realizing the direct *in vivo* application of constructs.

In the following section, we mainly enumerate the commonly used biodegradable polymers in indirect 3D bioprinting for blood vessels and vascularized tissue constructs. Table 2 summarizes the characteristics, combinations, and current applications of different scaffold and sacrificial materials.

**TABLE 2:** Common biomaterial inks for indirect 3D bioprinting covered in this review.

Biomaterials	Scaffold or sacrificial material	Natural or synthetic	Biodegradable or non-degradable	Biocompatibility	Mechanical property	Combinations	Applications	References
Gelatin	Scaffold and sacrificial material	Natural	Biodegradable	Good	Poor	PVA, Pluronic, agarose, HA, xanthan-gum	Earlobe-shaped channel system, liver model	Liu et al. (2008)
								Benton et al. (2009)
								Nichol et al. (2010)
								O’Bryan et al. (2017)
								Štumberger and Vihar, (2018)
Fibrin	Scaffold material	Natural	Biodegradable	Good	Poor	Gelatin, carbohydrate glass	Arteriole/venule	Hu et al. (2018)
								Schöneberg et al. (2018)
								Duarte Campos et al. (2020)
Alginate	Scaffold and sacrificial material	Natural	Biodegradable	Good	Medium	PVA, agarose, Pluronic F127, carbohydrate glass	Human heart- and kidney-like objects	Lee and Mooney, (2012)
								Rocca et al. (2018)
								Piras and Smith, (2020)
SF	Scaffold material	Natural	Biodegradable	Good	Good	Thermoplastic, plaster	Bone and cartilage engineering	Liu et al. (2013)
								Bidgoli et al. (2019)
PVA	Sacrificial material	Synthetic	Biodegradable	Good	Good	Gelatin, silk, agarose, alginate, fibrin, Matrigel, PLCL, PUU	100–1750 μm diameter channels	Tocchio et al. (2015)
								Hernández-Córdova et al. (2016)
								Hu et al. (2018)
								Im et al. (2018)
								Kim et al. (2018)
								Charron et al. (2019)
								Wu et al. (2019)
HA	Sacrificial material	Synthetic	Biodegradable	Good	Poor	Gelatin	Enzymatically digestible, 360–720 μm diameter channels	Thomas et al. (2020)
Agarose fiber	Sacrificial material	Natural	Biodegradable	Good	Good	Alginate, Gelatin	100–1,000 μm diameter channels	Bertassoni et al. (2014)
								Massa et al. (2017)
								López-Marcial et al. (2018)
Carbohydrate glass	Sacrificial material	Synthetic	Non-degradable	Cytotoxic when dissolved	Good	PEG, fibrin, alginate, agarose	150–750 μm diameter channels	Khattak et al. (2005)
								Miller et al. (2012)
Pluronic F127	Sacrificial material	Synthetic	Non-degradable	Cytotoxic	Good	Gelatin, sodium alginate, decellularized extracellular matrix	150–3,000 μm diameter channels	Homan et al. (2016)
								Daly et al. (2018)
								Ding and Chang, (2018)
								Hu et al. (2018)
								Xu et al. (2018)
								Yang et al. (2020)

### Scaffold Materials

Scaffold materials, whose purpose is to simulate the ECM and provide support for tissue regeneration in the human body, should have good biosafety, biocompatibility, biodegradability, and mechanical properties that are favorable for cell growth, proliferation, and migration (Lee A. Y. et al., 2014). Ideally, they should also be able to promote angiogenesis (Do et al., 2015; Xu et al., 2018). After crosslinking, scaffold materials need a certain degree of stiffness to maintain structural integrity during removal of sacrificial materials, and to support the flow of perfusion (Skylar-Scott et al., 2019). Controllable degradation rate consistent with the growth and repair rate of host tissue *in vivo* is also necessary (Malda et al., 2013; Serbo and Gerecht, 2013).

To date, various natural biodegradable polymers have been used for scaffold bioprinting, such as gelatin (Chen et al., 2012), fibrin (Schöneberg et al., 2018), and alginate (Jia et al., 2014); they are collectively referred to as hydrogels. They show excellent human ECM features and allow cell encapsulation (Malda et al., 2013), but they have all been deficient in some respect. Uncontrolled degradation and poor mechanical properties are the main problems, and they are also the research focus of material modification. The simplest and most used method is blending modification. In this approach, the hydrogel ratio is controlled to prevent excessive polymer concentration or dilution, which may have an adverse effect on cell behavior or mechanical properties (Malda et al., 2013; Duarte Campos et al., 2016; Zou et al., 2020).

Gelatin is one of the most widely used scaffold materials extracted from collagen, the main component of natural human ECM. Gelatin is composed of 85–92% protein, water, and mineral salts, and is highly susceptible to several proteases (Bello et al., 2020). Its excellent biocompatibility and similarity with collagen have made it the preferred material for the assembly of scaffolds. However, as a biomaterial ink, gelatin does have several drawbacks; these include low viscosity and yield stress, as well as relatively long crosslinking time, which leads to poor shape retention and structural collapse, and is the main obstacle to creating high resolution 3D pore or microchannel structures (O’Bryan et al., 2017). These disadvantages can be improved by making gelatin composites, or applying sacrificial materials to support the scaffold hydrogel before crosslinking (Moroni et al., 2018). In addition, the degradation rate of large solid gelatin *in vivo* is relatively slow (Daly et al., 2018). Large numbers of gelatin residues within thick tissues remain a challenge. Solutions may include lowering the degree of metacrylation and the macromer concentration (Nichol et al., 2010; Chen et al., 2012). Furthermore, by creating microchannels within the hydrogels to enhance host interaction, the degradation of gelatin can be enhanced (Daly et al., 2018). This is possibly because of the increased invasion by host immune cells, such as macrophages, which is vital for degrading gelatin hydrogels (Kim et al., 2014).

Gelatin-methacryloyl (GelMA) is the most commonly used material among gelatin derivatives and composites. GelMA is a photopolymerizable hydrogel made of gelatin derivatized with methacrylamide side groups (Van Den Bulcke et al., 2000). It has high biosafety compared with several gelatin-based hydrogels formed by chemical crosslinking methods, such as ones fabricated using glutaraldehyde or transglutimase derived from bacteria (Benton et al., 2009). GelMA is sensitive to matrix metalloproteinases and can be degraded by cells (Nichol et al., 2010). By adjusting the rate of polymerization and ratio of methacrylic acid, GelMA can maintain relatively good shape with adjustable mechanical properties (Benton et al., 2009). Research has shown that ECs as well as endothelial colony-forming cells can undergo active angiogenesis or vasculogenesis in GelMA hydrogels either *in vivo* or *in vitro* (Chen et al., 2012; Massa et al., 2017).

Fibrin scaffolds have a wide range of applications in tissue engineering, especially in bone tissue engineering. Fibrin is a natural biopolymer produced by thrombin cleavage of fibrinogen, and serves as a temporary scaffold for tissue healing in physiologic processes (Noori et al., 2017). It also plays an important role in specific receptor-mediated interactions with cells because of its ability to bind different types of proteins and growth factors, including FGF and VEGF that promote angiogenesis (Breen et al., 2009; Litvinov and Weisel, 2017). Fibrin hydrogel can be easily remodeled by ECs, which is favorable for fast angiogenesis. Nevertheless, similar to gelatin, fibrin rapidly degrades, and has poor mechanical stability, durability, and shape fidelity (Calderon et al., 2017). To overcome these problems, fibrin composites and mimics have been developed in 3D bioprinting. For example, by combining fibrin and gelatin, the stiffness of the hydrogel increased, and lower water loss on compression was observed (Schöneberg et al., 2018). Another attempt at a fibrin composite is called ELP-RGD, composed of ELP (elastin-like protein) hydrogel along with a cell adhesion RGD amino acid sequence derived from fibronectin. ELP hydrogel contains elastin (a kind of fibrin) -like repeat units alternating with biologically active domains (Madl et al., 2017). As a scaffold material, ELP-RGD has adjustable stiffness, is readily hydrolyzable with protease, and is able to promote matrix remodeling as well as cell proliferation (Chung et al., 2012). It has been demonstrated to be suitable for on-chip platforms with vascular-like networks (Duarte Campos et al., 2020).

Alginate is a natural polysaccharide extracted from alginic acid. The long polysaccharide chains provide it with pliability and gelling adeptness, and it undergoes hydrolytic cleavage under acidic conditions or enzymatic degradation by lyase (Pawar and Edgar, 2012; Rastogi and Kandasubramanian, 2019). Despite its mechanical instability and poor cell attachment, alginate is widely used as a hydrogel because of its low cost, good biosafety, and its ability to be rapidly but reversibly crosslinked by Ca^2+^ under mild conditions (Jia et al., 2016; Rastogi and Kandasubramanian, 2019). However, one important limitation of alginate application *in vivo* is the low degradation rate and unpredictable degradation process owing to the lack of alginate degrading enzyme in the human body (Reakasame and Boccaccini, 2018). Moreover, alginate shows relatively poor cell adhesion and infiltration (Balakrishnan et al., 2014). Measures like modification or mixing in additives, such as nanomaterials, peptides, and growth factors, are useful to regulate rheological properties, promote cell adhesion, or guide cell differentiation (Lee and Mooney, 2012; Piras and Smith, 2020). For example, by oxidative modification, more reactive sites are provided to the structure, accelerating the alginate’s biodegradability (Liang et al., 2011). Ino et al*.* combined sodium alginate with sacrificial molds of sugar structures, and by soaking the structure in a CaCl_2_ solution they achieved simultaneous dissolution of the mold and formation of calcium alginate hydrogel, thus simplifying and hastening the manufacturing process (Ino et al., 2020).

Silk fibroin (SF), a silk-derived protein-based material approved by the FDA (Perrone et al., 2014), has been used to make clinical sutures for many years. In recent decades, because of new processing techniques and further understanding of its properties, SF has attracted great interest in bone and cartilage engineering (Liu et al., 2013). Compared with the abovementioned materials, native silk fibers have excellent mechanical properties including good strength and toughness (Kundu et al., 2013; M et al., 2017). Other advantages of SF include good biosafety, good biocompatibility, controllable biodegradability and bone induction, and low immunogenicity (Wenk et al., 2009; Kundu et al., 2013). The host immune system has been shown to play a significant role in the degradation of SF scaffolds. Macrophages mediate the process, suggesting that SF is also bioresorbable (Wang et al., 2008). It is worth noting that SF remains strong during degradation, which is its unique advantage in tissue engineering (Kundu et al., 2013). Studies were carried out to produce vascularized scaffolds via SF. In combination with indirect 3D bioprinting, a layered SF-bioactive glass composite scaffold with excellent compressive strength, flexibility, and 10–50 μm micropores has been fabricated (Bidgoli et al., 2019). The scaffold comprises hierarchically micro and sub-micro pores, which are important features for promoting cell migration, differentiation, bone formation, and angiogenesis (Qi et al., 2018); results showed that the scaffold enhanced cell adhesion and cell proliferation (Bidgoli et al., 2019). However, drawbacks of SF such as lack of biological activity, general poor performance under humid conditions, and the difficulty of transportation and long-term storage may limit its further application. These limitations are potential future research directions.

Other scaffold materials commonly used in indirect 3D bioprinting but that are not yet, or rarely, used for vasculature fabrication include polyethylene glycol (PEG), hyaluronic acid (HA), PLA, PCL, poly (l-lactide-co-ɛ-caprolactone) (PLCL), and poly(lactic-co-glycolic) acid (PLGA) (Park et al., 2014; Houben et al., 2016; Aljohani et al., 2018; Im et al., 2018). Inorganic substances are more frequently used for osteochondral tissue fabrication because of their excellent mechanical properties. Future studies on these materials can be performed in the field of indirect 3D bioprinting for tissue vascularization.

### Sacrificial Materials

Ideal sacrificial materials should have good fluidity for free molding, rapid solidification to save printing time, a low expansion rate, and appropriate mechanical strength to achieve good shape fidelity. During the process of printing, there should be no adverse reactions with the scaffold material that result in deformation of the scaffold structure. If working in combination with scaffold materials containing cells or other bioactive factors, sacrificial materials should be chosen to ensure non-toxic and non-stimulatory conditions. Also, the conditions for their state transformation and removal should be mild and easy to achieve, preserving the shape and properties of the scaffold. When exposed to living cells *in vivo* or bioactive components, cytotoxicity, biocompatibility, and whether the removal conditions are inconducive are usually considered first.

Currently the commonly used sacrificial materials that come closest to meeting the conditions described earlier are carbohydrate glass (Miller et al., 2012), Pluronics (Afghah et al., 2020), and PVA (Zou et al., 2020). Carbohydrate glass and Pluronics are nonbiodegradable materials. Carbohydrate glass is a simple glass consisting of a mixture of carbohydrates, including glucose, sucrose, and dextran, and was one of the first materials applied to indirect 3D bioprinting as a sacrificial biomaterial ink (Miller et al., 2012). The synthetic glass shows sufficient mechanical stiffness to maintain its shape in air, as well as rapid dissolution to accelerate the process. Results demonstrated that it can be compatible with a variety of natural or synthetic hydrogel materials, such as agarose, alginate, fibrin, and Matrigel, adapting well to their different properties and means of crosslinking (Miller et al., 2012). Currently, vessels with diameters ranging from 150 μm to 1 mm and smooth in-plane junctions can be achieved with this sacrificial ink (Pollet et al., 2019). Pluronics are a class of amphiphilic tri-block copolymers popular in drug and clinical applications. Because they have the characteristics of solubilizer, emulsifier, and stabilizer, they are often used as excipients in pharmaceutical preparations (Jarak et al., 2020). The most frequently used Pluronic in tissue engineering is Pluronic F127, which can rapidly dissolve in aqueous media or biological fluids. Its sol-gel transition at room temperature and convenient removal attracts attention as a sacrificial ink. Channels with a diameter as small as 150 μm can now be printed with Pluronic F127 (Homan et al., 2016). However, both carbohydrate glass and Pluronic F127 show cytotoxicity when dissolved, which is one of the most prominent shortcomings (Miller et al., 2012; Deng et al., 2020). Besides, Pluronic F127 liquifies at low temperatures, making it difficult to use with some scaffold materials that require these temperatures during casting, such as collagen and matrix gelatin (Ding and Chang, 2018; Hu et al., 2018).

As mentioned above, nonbiodegradable materials usually exhibit certain cytotoxicity and their removal is relatively cumbersome. As a result, researchers have investigated biodegradable materials, which show higher biosafety and are expected to achieve direct application *in vivo*.

PVA, a commonly used biodegradable sacrificial material, has satisfactory biocompatibility and similar functions to natural tissues, including high water content, high elasticity, and low interfacial tension with biological fluids (Teodorescu et al., 2019). It shows resistance to protein absorption, which is important for bone formation (Kim et al., 2018). The ease of printability allows it to be used for repeatable fabrication of complex vascular patterns (Hu et al., 2018). In terms of mechanical properties, PVA has good strength and stiffness at 25°C (Charron et al., 2019) and fits well with different biodegradable natural polymers, such as gelatin and silk (Mohanty et al., 2016). Tocchio et al*.* used PVA successfully to make sacrificial templates with characteristic sizes of 100–500 μm in multi-branch structures, which helped to further simulate the complex environment of cell growth, and showed great potential for production of large-sized vascularized scaffolds that would meet clinical needs (Tocchio et al., 2015). Compared to other sacrificial materials, the stable chemical properties, convenience of preservation, and low cost of PVA are huge advantages for its use in industrial production. However, owing to the lack of bioactive components, PVA tends to be resistant to protein absorption and cell adhesion, which limits its further application outside bone tissue engineering (Schmedlen et al., 2002). Furthermore, if PVA filaments are too large in diameter (>500 μm reported), they are likely to deform as they cannot support their own weight (Hernández-Córdova et al., 2016). More in-depth research is needed in the future.

HA-based enzymatically degradable photoink was developed by different research teams (Zhu et al., 2017; Thomas et al., 2020). HA is a linear polysaccharide. It is an essential component of the ECM and has vital effects on many cellular responses, including cellular signaling, wound repair, morphogenesis, and angiogenesis (Burdick and Prestwich, 2011). In the abovementioned two experiments, HA was chemically modified to achieve photopolymerization and mixed with gelatin to form the photoink. The hydrogels could be digested with hyaluronidase to achieve fast (within hours) and collaborative (not limited by graft size) degradation. Channels ranging from 50 to 720 µm have been successfully fabricated and achieved vascularization. However, the enzymolysis process may reduce EC activity, and because the enzyme is encapsulated in the bioink, degradation occurs at the same time as printing, which exerts a certain amount of time pressure (Zhu et al., 2017; Thomas et al., 2020).

Other research teams have also produced alternate solutions; for example, agarose was used as a sacrificial ink (Massa et al., 2017). It shows good rheological properties and printability similar to Pluronics when combined with alginate (López-Marcial et al., 2018). Currently, agarose has been shown capable of fabricating 100–1,000 μm diameter pipes and forming a smooth channel surface for cell inoculation (Massa et al., 2017). Another example is gelatin, that has been used as a sacrificial material and is removed by warming (Skylar-Scott et al., 2019). Channels over 400 μm in diameter could be printed with high fidelity. Another team used alginate and CaCl_2_ to create ultrafine fibers with a size range of 150–200 μm, and clear, interconnected microchannel structures were observed through the hydrogel as a result (Hammer et al., 2014). It is worth mentioning that some of the removal processes of these biodegradable sacrificial materials are physical processes. The utilization of their biodegradable properties needs to be further explored, as they show great potential to facilitate simplified fabrication processes and direct *in vivo* application of tissue constructs.

### Cells and Biological Factors

When 3D printing techniques print with cells, the printing ink is also referred to as bioink. It includes cells, biomaterials that serve as a cell-delivery medium, and biological factors (Groll et al., 2018). The existence of cells and biological factors require higher biosafety of the materials to maintain good bioactivity of the bioinks as discussed before. Also, to prevent cells from excessive shear forces, low viscosity fluid is required. Viscosity and rheological properties greatly influence the printability of the bioinks, and they are mainly determined by the molecular weight and concentration of polymer in solution (Schwab et al., 2020). Gels with shear-thinning properties or solutions containing hydrogel precursors are preferred. In current studies of indirect 3D bioprinting for vascular systems, HUVECs, smooth muscle cells (SMCs), and fibroblasts have been used, and the cell viability is generally over 80% with high cell density (Table 3). Still, more efforts should be put on developing optimized bioinks in future investigations.

**TABLE 3:** Summary of indirect 3D bioprinting applications and bioink selection for different tissue vascularization covered in this review.

Category	Sub-category	Sacrificial material	Scaffold material	Cells and cell density	Cell viability	Progress	Limitations	References
Vascular grafts	Arteriole/venule	Gelatin	Fibrin and collagen/fibrin blends	HUVECs (∼10^7^ cells/ml); SMCs (∼10^6^ cells/ml); normal human dermal fibroblasts (—)	∼83%/91% (1d/4d, SMCs)	*In vitro* model success	Unable to meet human transplantation standards	Schöneberg et al. (2018)
	Branched vascular structure	Pluronic-nanoclay	Alginate	—	—	*In vitro* non-cell model success	No biological function	Afghah et al. (2020)
Highly vascularized tissue	Heart-like structure	Pluronic F127	Alginate	—	—	Simplified models for conceptual validation	No good method to fabricate complex structures	Zou et al. (2020)
	Valentine-shaped heart	PVA	Alginate and agarose	HUVECs (∼10^6^ cells/mL); H9c2 rat myoblasts (∼10^6^ cells/ml)	∼95%/90% (1d/14d)	A hollow structure containing a network of micro-fluid channels	Difficult to imitate the ultrastructure of capillaries; low degree of simulation	Zou et al. (2020)
	Simplified cardiac scaffolds	PVA	PUU	Primary human cardiac myocytes (∼10^4^ cells/scaffold)	94% (1d)	A perfusable scaffold with mechanical properties similar to cardiac tissue, and good biocompatibility with cardiac myocytes	A geometrically simplified *in vitro* scaffold mainly for material performance test	Hernández-Córdova et al. (2016)
	Cardiac spheroids	Gelatin	Collagen I and Matrigel	Cardiomyocytes with primary cardiac fibroblasts (∼10^9^ cells/ml in total); HUVECs (∼10^7^ cells/ml)	Enhanced cell viability throughout the bulk tissue compared to nonvascular tissue	A perfusable cardiac tissue that fuses and beats synchronously over a 7-day period with high cellular density	Lack of sufficient microvascular network formation; a modest contractility (∼1% strain) only	Skylar-Scott et al. (2019)
	Gut-like tissue fragments	PVA	Matrigel, gelatin, and fibrin	Caco-2 intestinal epithelial cells; HUVECs (∼10^7^ cells/ml)	Good cell co-culture results	An *in vitro* gut model capable of sustaining cells long term	A simplified model mainly for conceptual validation	Hu et al. (2018)
	Liver tissue model	Agarose fiber	GelMA	HUVECs (∼10^5^ cells/ml); HepG2/C3A cells (∼10^6^ cells/ml)	>80% (2d)	A vascularized liver tissue model for mimicking *in vivo* conditions and testing drug diffusion and toxicity	Difficult to imitate the ultrastructure of capillaries	Massa et al. (2017)
	Liver tissue fragments	PVA and PLA	Gelatin	Liver hepatocellular carcinoma (HepG2) cells (∼10^6^–10^8^ cells/ml)	Good HepG2 cell proliferation to a high cell density	A perfusable thick engineered construct with cellular densities of native tissues	A simplified model for conceptual validation; difficult to create channels with diameter <1 mm	Pimentel et al. (2018)
	Renal proximal tubule models	Pluronic F127	Gelatin	Proximal tubule epithelial cells (∼10^7^ cells/ml); glomerular microvascular epithelial cells (—)	Healthy cell phenotype was observed	A 3D vascularized proximal tubule model that can be independently addressed to investigate renal reabsorption	The reabsorptive properties may be improved by reducing the proximal tubule lumen diameter and the separation distance between the proximal tubule and vascular conduits	Lin et al. (2019)
	Kidney-like structure	Pluronic F127	Alginate	—	—	Simplified models for conceptual validation	No good method to fabricate 3D highly vascularized network in thick tissue or organ	Rocca et al. (2018)
Vascularized osteochondral tissue	Cartilage tissues	Pluronic F127	GelMA	Bone marrow derived mesenchymal stem cells (∼10^7^ cells/ml)	Cells remained viable after 24 h	A promising approach for guiding vascularization and implant remodeling during endochondral bone repair	No obvious enhanced overall-level bone formation	Daly et al. (2018)
Vascularized skin	Finger-shaped highly elastic scaffold	PVA	PLCL	Human dermal fibroblasts (∼10^6^ cells/ml)	Considerable collagen and new blood vessels were observed at 4 weeks	A customized scaffold successful in animal experiments and may act as a dermis substitute	A simplified model without hierarchical structure	Im et al. (2018)
	Thermoresponsive ‘stiffness memory’ elastomeric nanohybrid scaffolds	PVA	PUU-POSS	3T3-J2 mouse embryonic dermal fibroblasts (∼10^4^ cells/scaffold)	Good ingrowth of tissue and new blood vessels were observed at 4 weeks	A unique smart elastomer scaffold that can guide the growth of myofibroblasts, collagen fibers, and blood vessels at real 3D scales	Slow ingrowth of host blood capillaries; local inflammatory response	Wu et al. (2019)

## Preclinical and Clinical Applications of Indirect 3D Printing for Different Tissue Vascularization and Biodegradable Ink Selection

Nowadays, only a few tissues with less stringent vascular structures, such as cartilage and the cornea, have achieved good clinical outcomes (Rouwkema et al., 2010; Seifu and Mequanint, 2012; Pimentel et al., 2018). Advanced techniques and suitable biomaterials are essential for the development of functional engineered tissues. Indirect 3D bioprinting and the related biodegradable inks provide new opportunities for commercial development. The process has been applied to manufacturing various tissues and has achieved corresponding results. In general, our appraisal can be divided into vascular grafts and vascularized tissue; the latter includes highly vascularized tissue, vascularized osteochondral tissue, and vascularized skin, as shown in Table 3.

### Vascular Grafts

**FIGURE 2:**
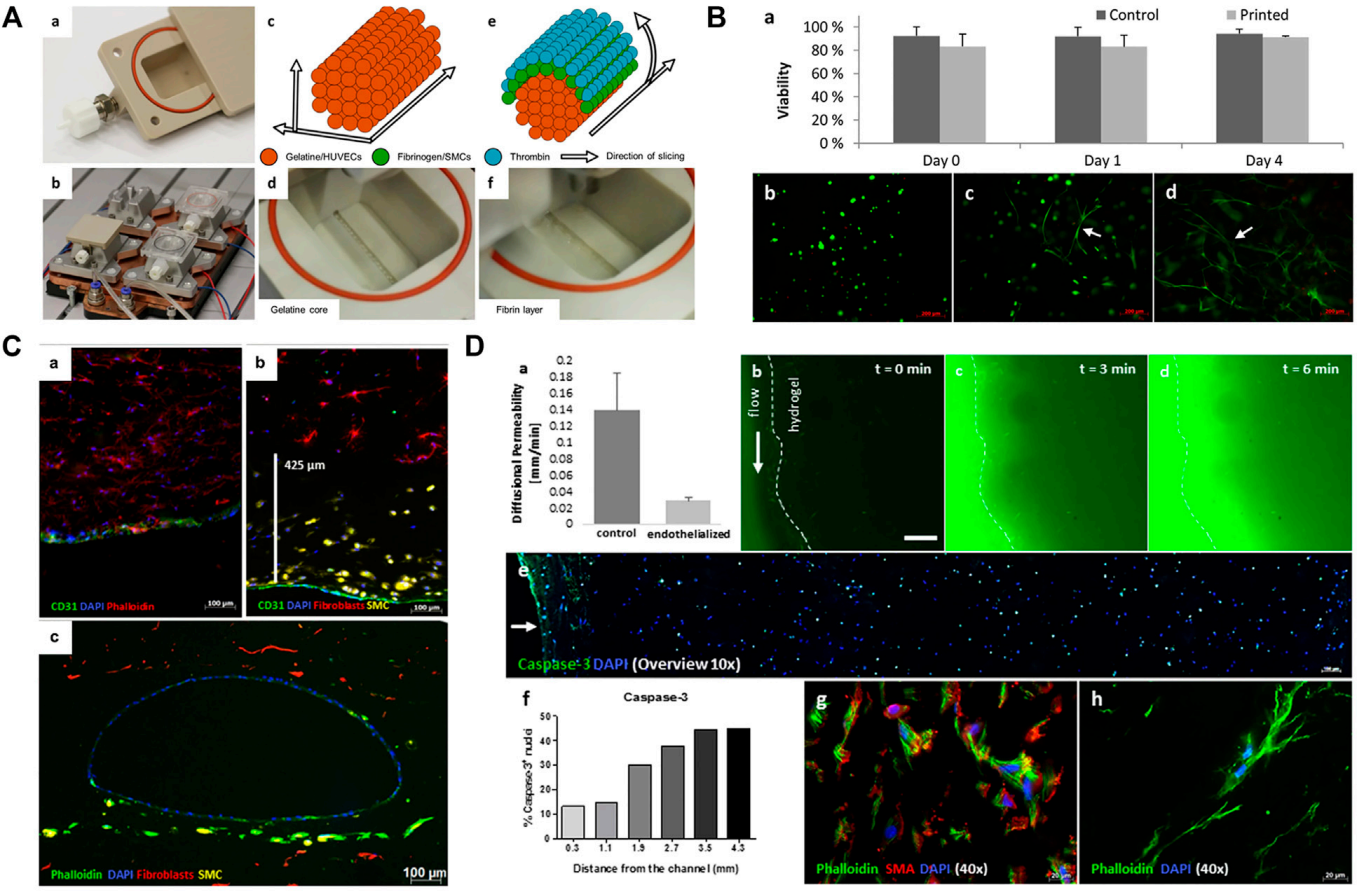
Printing of blood vessels with multi-layered structure. **(A)** Schematic diagram showing the printing procedure in a bioreactor. The printing includes a gelatin core and a surrounding fibrin layer. **(B)** Results showed that cell viability was not affected after the printing process. **(C)** Fluorescence micrographs show the homogenous distribution and good combination of ECs, SMCs, and fibroblasts. **(D)** Permeability testing and cell viability evaluation. Adapted with permission (Schöneberg et al., 2018). Copyright 2018, Nature Publishing Group.

Most vascular regions treated during surgery are larger than 1 mm in diameter, which, theoretically, both direct and indirect 3D bioprinting can achieve. Biomaterial selection requirements for tissue-engineered vascular grafts are low immunogenicity, good mechanical properties, and similarity to native tissue characteristics (Liu et al., 2018). Compared with non-degradable polymers, biodegradable natural polymers (such as decellularized tissue scaffolds and fibrin) have relatively poor mechanical properties, but they have lower antigenicity and can better simulate natural tissue structures (Aper et al., 2016). Meanwhile, biodegradable synthetic polymers (such as PGA and PCL) can have adjustable mechanical properties and degradation rate, and they are considered the ideal biomaterials for tissue-engineered vascular grafts (Liu et al., 2018). Afghah et al*.* used embedded extrusion bioprinting with a composite Pluronic-nanoclay support-bath and biocompatible alginate to create a branched vascular structure with diameters of several millimeters. This vascular mold showed good mechanical properties and preservation of shape fidelity after removal from the support-bath, but its biological functions have not yet been verified (Afghah et al., 2020). While Schöneberg et al. used the indirect bioprinting technique to fabricate biofunctional multi-layered blood vessel models *in vitro*, which direct printing cannot yet achieve (Schöneberg et al., 2018). They used three different degradable hydrogels with three different cell types to simulate and reconstruct the adventitia (fibroblast matrix), medial layer (elastic SMC), and intima (endothelium), and successfully replicate the three-layered natural vascular channel structure with a wall thickness of up to 425 μm, and diameters of around 1 mm (Figure 2). These bioinks provide a friendly living environment for cells. Currently, engineered vascular grafts do not meet human transplantation standards, and most of them are used for *in vitro* experiments, such as drug prescreening or preliminary concept verification.

### Highly Vascularized Tissue

Currently, most studies are in the conceptual validation phase and, to date, there are no good methods or compatible bioinks to fabricate a 3D functional, highly vascularized network in thick tissue or organ constructs, which limits the development of tissue engineering (Duan, 2017).

For highly vascularized tissue, such as liver, kidney, and heart, more precise microvessels are required, ranging from a few microns to millimeters. However, the current techniques have difficulty achieving this accuracy and resolution. Zou et al. created a valentine-shaped synthetic heart with a simplified aorta by using indirect 3D bioprinting and biodegradable alginate (Zou et al., 2020). They demonstrated no collapse of the scaffold structure and 90% cell viability. Nevertheless, the constructed microchannels are still at the level of hundreds of microns, and it remains difficult to emulate the ultrastructure of capillaries. An *in vitro* liver model (GelMA loaded with HepG2/C3A) was developed to test drug toxicity (Massa et al., 2017), and 3D human heart- and kidney-like objects, composed of dozens of alginate layers, were produced by embedded extrusion bioprinting (Rocca et al., 2018); however, these models were about 2 cm^3^ in size, with very simple vascular structure, and were used only for concept validation. Lin et al*.* used indirect 3D bioprinting to fabricate adjacent open cavities (representing proximal renal tubules and parallel blood vessels) embedded in the permeable ECM. After endothelialization and epithelization, the selective reabsorption and vectorial transmission of solute were realized through external circulation devices, which favor the further study of tissue-engineered kidneys (Lin et al., 2019).

**FIGURE 3:**
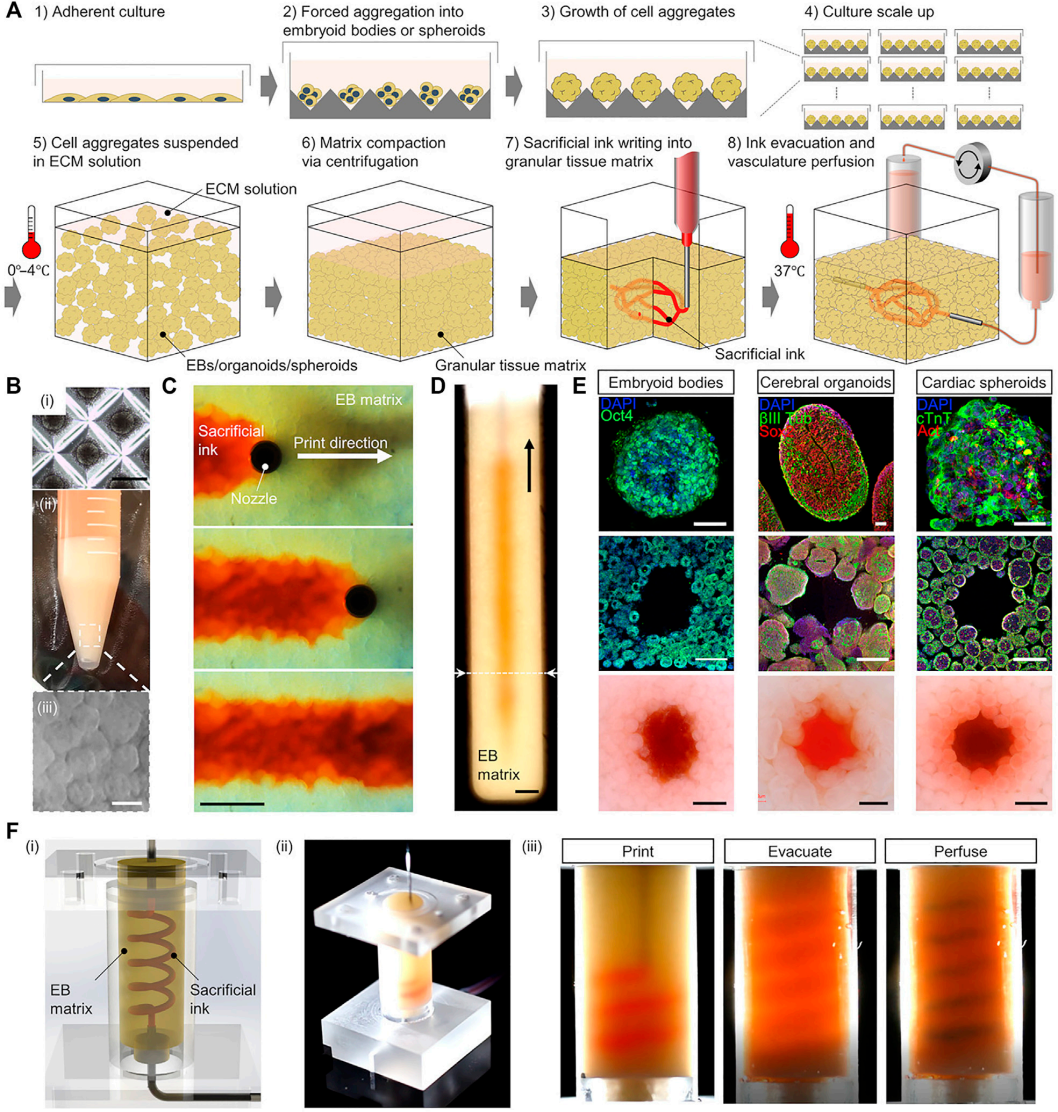
Printing of highly vascularized tissues with high cell density. **(A)** Schematic diagram of the indirect 3D bioprinting workflow. **(B)** Organ building block (OBB) tissue matrix formation. **(C,D)** Sacrificial ink writing within an embryoid body (EB) matrix. **(E)** Examples of different OBB-based matrices. **(F)** Fabrication of a helical vascular structure in an EB matrix. Reproduced with permission (Skylar-Scott et al., 2019). Copyright 2019, American Association for the Advancement of Science (AAAS).

There are also some special needs for biomaterials. Highly vascularized tissues usually consist of large amounts of active cells, which require the biomaterials to be sufficiently friendly to cells and cell migration, growth, and proliferation, and should facilitate substance exchange (Xie et al., 2020). Usually, softer biomaterials are needed; indeed, one important requirement for cardiac scaffolds is that it should not provide resistance to muscle contraction during systole while providing mechanical support to resist the tensile stress during diastole (Hernández-Córdova et al., 2016). This requires the elastic modulus of the biomaterial to match that of the myocardial reported interval (7.9–1,200 kPa) (Courtney et al., 2006; Chan et al., 2013; Hernández-Córdova et al., 2016). Alginate, gelatin, fibrin, and collagen can be used for cardiac scaffolds (Alonzo et al., 2019). In an indirect 3D bioprinting case, PUU was developed as a scaffold material that had suitable elastic modulus. They also used PVA as sacrificial material, and finally created a cardiac scaffold containing 300–500 µm channels. The biomaterial showed good biocompatibility with cardiomyocytes (Hernández-Córdova et al., 2016). Meanwhile, to verify the feasibility of multicellular tissue printing, Skylar-Scott et al*.* reported an embedded indirect 3D bioprinting method to create perfusable vascular channels in ECM solution with organ building blocks (OBBs) composed of thousands of patient-specific-induced pluripotent stem cell–derived organoids, as shown in Figure 3. As an example, they then fabricated a cardiac tissue with physiological functions over a 7-day period with high cellular density, showing the huge prospects of indirect 3D bioprinting (Skylar-Scott et al., 2019).

### Vascularized Osteochondral Tissue

The 3D printing technique is relatively mature in the field of osteochondral tissue engineering and has been used in the fabrication of long bones, mandibles, cheekbones, human finger bones, and other structures (Hollister et al., 2005; Lee et al., 2005; Wang et al., 2009). Biomaterials for osteochondral tissue engineering emphasize mechanical properties (Daly et al., 2017). For the design and production of vascularized bone tissue engineering scaffolds, pore-forming agents are usually added to form pores of specific sizes (>300 μm) to promote angiogenesis (Karageorgiou and Kaplan, 2005; Wang J.-Q. et al., 2019; Gonzalez-Fernandez et al., 2019). Uniform channels of controlled size that simulate the natural morphology of bone tissue, including Volkman’s and Haversian canals for better osteogenesis, are more and more created in molds via indirect 3D bioprinting (Houben et al., 2016; Houben et al., 2017). However, few studies have focused on the analysis of angiogenesis and new bioink development.

For endochondral bone repair, current techniques enable *in vivo* angiogenesis around cartilage models (Thompson et al., 2016), but the internal regions of the models remain nonvascular (Mesallati et al., 2015). Daly et al*.* constructed a microchanneled cartilage template using sacrificial Pluronic ink. After *in vivo* cultivation, they found that, compared to solid templates, channeled templates showed better vascularization, more degradation of cartilage precursor hydrogel in the core region, less ectopic bone formation, and better integration with the host tissue, which are all clinically important (Daly et al., 2018). However, more evidence is needed to demonstrate a difference in total bone formation between the channeled and solid templates for endochondral bone repair.

### Vascularized Skin

Creating man-made skin grafts for wounds and burn healing is the primary purpose of skin tissue engineering (Adams and Ramsey, 2005). Tissue-engineered skin grafts should be non-toxic, have low inflammatory response, allow water vapor transmission, and act as a barrier. They are also expected to quickly adhere to the wound surface, have controllable degradation, and promote angiogenesis (MacNeil, 2007). Biodegradable, non-toxic, non-immunogenic, and non-inflammatory biomaterials with low risk of disease transmission and easy access are ideal skin substitutes (Vig et al., 2017). To date, indirect 3D bioprinting has not been widely applied in vascularized skin tissue fabrication. Wu et al. created a thermoresponsive stiffness memory elastomer nanohybrid scaffold via indirect 3D bioprinting and found that the scaffold promoted fibroblast proliferation *in vitro* and angiogenesis *in vivo* (Wu et al., 2019)*.* Another team found that by combining bioactive peptide hydrogels with scaffolds having finger-shaped pores created via indirect 3D bioprinting, more vessels and more collagen I and III formed in the scaffolds (Im et al., 2018). Most studies that focused on tissue design have been based on simplified skin models, while few models consider controlled porosity, biodegradable material selection, and cell distribution (Lee V. et al., 2014; Yan et al., 2018). It is important to mention that for the reconstruction of simple epidermis or thin dermis, vascularization is not necessary.

## Conclusion

As described in this paper, tissue vascularization has always been a critical issue in tissue engineering and is key to the application and survival of engineered tissue constructs *in vivo*. Many accomplishments have demonstrated the feasibility of indirect 3D bioprinting for manufacturing blood vessels and vascularized tissue constructs, and most experiments show that indirect 3D bioprinting has advantages in channel structure design and construction. On the other hand, the selection of biodegradable inks is an important aspect of indirect 3D bioprinting for successful 3D tissue construct fabrication and *in vitro/vivo* application. Theoretically, indirect 3D bioprinting allows a wider range of materials to be used, since the use of sacrificial materials lowers the mechanical performance requirements for scaffold materials. However, this introduces new requirements for the sacrificial materials. At present, owing to the lack of satisfactory biodegradable materials, further *in vivo* applications are limited. A large number of studies are investigating advanced sacrificial/scaffold bioinks, which assists in the assembly of biodegradable, biosafe, bioactive, and more bionic structures at higher resolution. Efforts are now transitioning from theoretical verification to tissue and organ model construction. We expect that with the future continuous development of biodegradable materials, the use of indirect 3D bioprinting will continue to increase and will contribute to the field of tissue engineering.
